# Characterization of Contaminants from a Sanitized Milk Processing Plant

**DOI:** 10.1371/journal.pone.0040189

**Published:** 2012-06-28

**Authors:** Sara Cleto, Sónia Matos, Leon Kluskens, Maria João Vieira

**Affiliations:** IBB-Institute for Biotechnology and Bioengineering, Centre of Biological Engineering, Universidade do Minho, Campus de Gualtar, Braga, Portugal; The Scripps Research Institute, United States of America

## Abstract

Milk processing lines offer a wide variety of microenvironments where a diversity of microorganisms can proliferate. We sampled crevices and junctions where, due to deficient reach by typical sanitizing procedures, bacteria can survive and establish biofilms. The sampling sites were the holding cell, cold storage tank, pasteurizer and storage tank - transfer pump junction. The culturable bacteria that were isolated after the sanitation procedure were predominantly *Pseudomonas* spp., *Serratia* spp, *Staphylococcus sciuri* and *Stenotrophomonas maltophilia*. We assayed several phenotypic characteristics such as the ability to secrete enzymes and siderophores, as well as the capacity of the strains to form biofilms that might contribute to their survival in a mixed species environment. The *Pseudomonas* spp. isolates were found to either produce proteases or lecithinases at high levels. Interestingly, protease production showed an inverse correlation with siderophore production. Furthermore, all of the *Serratia* spp. isolates were strong biofilm formers and spoilage enzymes producers. The organisms identified were not mere contaminants, but also producers of proteins with the potential to lower the quality and shelf-life of milk. In addition, we found that a considerable number of the *Serratia* and *Pseudomonas* spp. isolated from the pasteurizer were capable of secreting compounds with antimicrobial properties.

## Introduction

One of the most challenging tasks in any industrial food processing line is the upkeep of sanitary conditions as the high availability of nutrients and oxygen favour microbial growth. Furthermore, the actual set-up of the processing line - including crevices, valves and gaskets - makes proper disinfection by regular sanitation procedures more difficult and less efficient. Residues inherent to the processing of food accumulate in these locations and provide a setting prone to colonization by bacteria, especially in the form of biofilms.

Bacteria can adhere to surfaces and once that attachment becomes irreversible, biofilm starts forming. Bacteria form biofilms by encasing themselves in a protective and unifying matrix composed of exopolysaccharides (EPS), proteins, and often DNA. Bacteria in biofilms are more resistant to the action of sanitizing agents than are their planktonic counterparts [1], [2]. This increased resistance is likely multifaceted, arising from inherent differences in the physiological states of bacteria within the biofilm as well as from the protective nature of the matrix. Thus, biofilms are crucial sources of contamination [3]–[5]. In natural settings, most biofilms comprise multiple species. Even bacteria not usually capable of adhering to a surface and secreting matrix can persist within the biofilm community by adhering to the matrix produced by others. In addition to allowing bacteria to persist, biofilm communities also contribute to the corrosion of surfaces and piping [6], [7].

Both the existence of biofilms and the persistence of microorganisms in processing lines is prevalent [7]–[12]. Yet, this issue was until recently, not specifically addressed in sanitation plans under Hazard Analysis and Critical Control Point (HACCP) regulations, more specifically under its Principle 2 of determination of the critical control points. Such points consist of locations of potential contamination of the product [13].

Studies screening for the presence of microbes before and after sanitation procedures revealed that many organisms survive harsh treatments [14]. Despite a lack of direct evidence, increased resistance due to biofilms has been proposed as an explanation for the persistence of microorganisms post-sanitation [14]–[16].

The most common shelf-life limiting organisms found in dairy processing plants are species belonging to the *Pseudomonas* genus, such as *P. fluorescens* and *P. putida* [17]–[19]. These microorganisms are the most commonly found in refrigerated milk and have the ability to grow under refrigeration as well as to produce a number of heat-stable spoilage enzymes [19]. Degradation by these enzymes directly impacts the organoleptic properties of the final product, limiting the shelf life of processed milk. Antibiotic-resistant *Staphylococcus* spp. have also been recurrently found in some milk processing lines in North America [18], [20]. The presence of *Staphylococci* was attributed to contamination from the animal itself, due to chronic mastitis. Normal animal commensals such as *Enterococci* and *Lactobacilli* have previously been described within a milk farm, but apparently these did not arise from bovine origin [21].

In this study, we aimed to isolate organisms from various surfaces of a milk processing line in order to characterize the diversity of organisms there found. We also sought to characterize these microorganisms in terms of their spoilage potential, their capacity to adhere to surfaces in the form of biofilms and - very importantly - to establish whether the isolates were capable of secreting compounds with antimicrobial activity against other bacteria.

## Results

**Figure 1:**
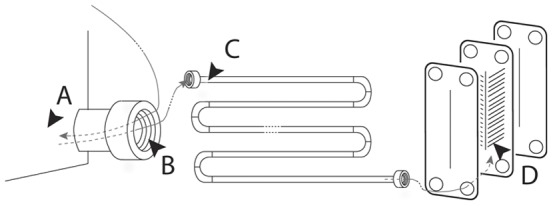
Diagram of devices and sites sampled within the milk processing plant. Samples were collect from the storage tank (A), from the junction between the storage tank and the transfer pump (B), from the holding cell (C) and from the inner part of the pasteurizer (D), as indicated by the black arrow heads. Grey arrows indicate the flow of milk (large grey dashes indicate inside locations) and the small black dashed lines indicate the simplification of the processing line organization. The devices are not designed to scale.

**Figure 2:**
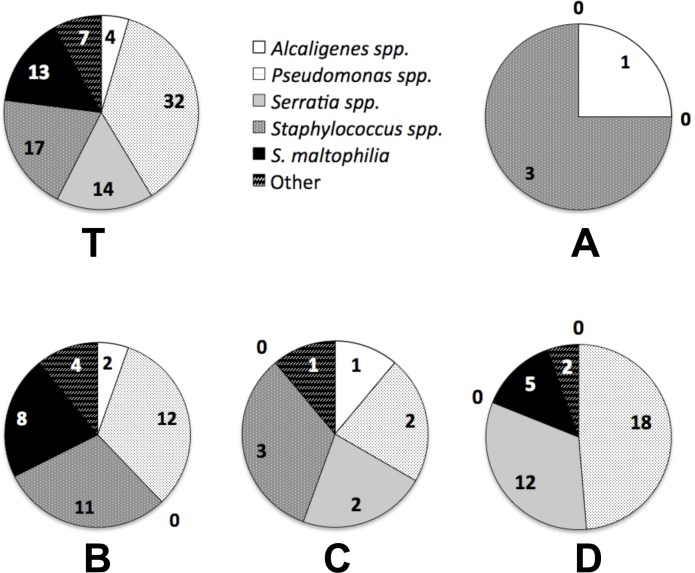
Microbial isolate profile from a sanitized milk processing line. Microorganisms isolates recovered in total from the sampling sites (T) distributed by location: storage tank (A), junction between the storage tank and the transfer pump (B), holding cell (C) and inner part of the pasteurizer (D). Microorganisms belonging to the genera *Achromobacter, Brevibacterium, Cupriavidus, Ochrobactrum, Raoultella* and *Rhodococcus* were listed as "other". The number of isolates is indicated within each pie segment. (T) Total isolates obtained from all samples organized by genus; (A–D) Breakdown of isolate distribution per sampling site.

The culturable isolates recovered from the crevices of cleaned devices from a milk processing plant (Fig. 1) and identified by 16S rDNA included five prevalent groups of bacteria in total: *Pseudomonas* spp. (37%); *Staphylococcus* (20%); *Serratia* spp. (16%), *Stenotrophomonas* sp. (15%), and *Alcaligenes* (5%). Microorganisms belonging to the genera *Achromobacter, Brevibacterium, Ochrobactrum, Raoultella* and *Rhodococcus* were also isolated (Fig. 2T). Of the four sampling sites, the pasteurizer and junction between the storage tank - transfer pump (junction) yielded 37 isolates each, while the holding cell had 9 isolates and the storage tank yielded only 4 culturable isolates in the conditions used. The sample originating in the pasteurizer (heat plates) predominantly contained *Pseudomonas* spp. and *Serratia* spp., whereas the junction was mainly dominated by *Pseudomonas* spp., *Staphylococcus* spp. and *Stenotrophomonas* sp. isolates. *Serratia* spp. were only isolated from the pasteurizer and holding cell (Fig. 2A-D).

All isolates were screened for their capacity to produce compounds that induce changes in milk properties, such as flavour, texture and smell. These properties have a direct impact on the commercial value of the final product. The production of the three most relevant classes of such compounds - proteases and lipases and lecithinases - was assessed by plating on appropriate medium as described in the materials and methods. Forty-six percent of the total isolates were incapable of growing on the protease detection medium (Table 1). Most organisms that were capable of growing on such media were also able to secrete proteases at high levels (46%), whereas only 8% displayed little or no activity (Table 1). The group with the largest number of producers was *Pseudomonas* spp. with 13 high-producing isolates, followed by *Serratia* and *Stenotrophomonas* at 9 isolates each. For *Stenotrophomonas* and *Staphylococcus* spp., any organisms that were capable of growing also produced high levels of proteases. *Pseudomonas* spp. also stood out as the genus with isolates most capable of producing lecithinases, while all other isolates were poor producers (Table 2). Furthermore, it was also observed that the isolates belonging to the genus *Pseudomonas* in general either produced high levels of proteases or of lecithinases (Table S1). Under the test conditions, the production of lipases was very low and restricted to one *Serratia* isolate and two *Staphylococcus* isolates (data not shown).

**Table 1:** Protease production by milk processing plant isolates.

	Total Isolates	*Pseudomonas*	*Serratia*	*Staphylococcus*	*Stenotrophomonas*	*Alcaligenes*	Other
**0/+**	**8 (7)**	3 (1)	7 (1)	0	0	50 (2)	43 (3)
**++/+++**	**46 (40)**	41 (13)	64 (9)	35 (6)	69 (9)	50 (2)	14 (1)
**ND**	**46 (40)**	56 (18)	13 (4)	65 (11)	31 (4)	0	43 (3)

The percentage of isolates within each genus exhibiting a given phenotype is shown with the number of isolates tested in parentheses. Little or no production (0/+), medium to high production (++/+++), and no growth on assay medium (ND).

**Table 2:** Lecithinase production by milk processing plant isolates.

	Total Isolates	*Pseudomonas*	*Serratia*	*Staphylococcus*	*Stenotrophomonas*	*Alcaligenes*	Other
**0/+**	**32 (28)**	3 (1)	43 (6)	76 (13)	15 (2)	25 (1)	71 (5)
**++/+++**	**22 (19)**	34 (11)	14 (2)	24 (4)	0	25 (1)	14 (1)
**ND**	**46 (40)**	63 (20)	43 (6)	0	85 (11)	50 (2)	14 (1)

The percentage of isolates within each genus exhibiting a given phenotype is shown with the number of isolates tested in parentheses. Little or no production (0/+), medium to high production (++/+++), and no growth on assay medium (ND).

To begin to define characteristics of the isolates that may enable them to survive in the processing line despite regular, well-established, cleaning protocols, we next assessed the ability of these organisms to produce compounds that may confer a selective advantage in the face of poor growth conditions. Iron availability is essential for an organism to survive and siderophore production allows organisms to sequester iron from their environment [22]–[24]. Therefore we assessed the isolates for their ability to produce siderophores using a CAS agar assay (Table 3). Of the 37 isolates that produced high levels of iron chelators, 20 were from the *Pseudomonas* isolates and 9 were *Serratia* spp. Notably, none of the 11 *Stenotrophomonas* isolates that grew on CAS plates were able to produce siderophores at high levels (Table 3).

**Table 3:** Siderophore secretion by milk processing plant isolates.

	Total Isolates	*Pseudomonas*	*Serratia*	*Staphylococcus*	*Stenotrophomonas*	*Alcaligenes*	Other
**0/+**	**48 (42)**	31 (10)	29 (4)	59 (10)	85 (11)	25 (1)	86 (6)
**++/+++**	**43 (37)**	63 (20)	64 (9)	24 (4)	0	75 (3)	14 (1)
**ND**	**10 (9)**	6 (2)	3 (1)	18 (3)	15 (2)	0	14 (1)

The percentage of isolates within each genus exhibiting a given phenotype is shown with the number of isolates tested in parentheses. Little or no production (0/+), medium to high production (++/+++), and no growth on assay medium (ND).

Another feature of the isolates that could provide them with a selective advantage is the ability to produce antimicrobial compounds. Thus, we assessed growth inhibition of lawns of *Escherichia coli* CECT 434 or *Staphylococcus aureus* CECT 976. The majority of the organisms (82%) did not produce detectable levels of antimicrobial compounds against the tested organisms (Table 4). Only isolates from the *Pseudomonas* and *Serratia* genera produced any detectable activity. More than 80% of the producing organisms were isolates derived from the pasteurizer (Table S1; Table 4).

**Table 4:** Antimicrobial production by milk processing plant isolates against *E. coli* or *S. aureus*.

	Total Isolates	*Pseudomonas*	*Serratia*	*Staphylococcus*	*Stenotrophomonas*	*Alcaligenes*	Other
N	82 (71)	69 (22)	57 (8)	100 (17)	100 (13)	100 (4)	100 (7)
P	18 (16)	31 (10)	43 (6)	0	0	0	0

The percentage of isolates within each genus exhibiting a given phenotype is shown with the number of isolates tested in parentheses. Antimicrobials were produced (P), or not produced (N).

To examine whether the isolates are able to survive within the sampling site by formation of biofilms, we assessed the ability of each isolate to adhere to surfaces. Adherence is the first step required for biofilm formation. Perhaps not surprisingly, over 70% of the isolates were strong adherers to polyethylene (Table 5).

**Table 5:** Milk processing plant isolates adherence.

	Total Isolates	*Pseudomonas*	*Serratia*	*Staphylococcus*	*Stenotrophomonas*	*Alcaligenes*	Other
**0/+**	**29 (25)**	47 (15)	0	35 (6)	8 (1)	25 (1)	71 (5)
**++/+++**	**71 (62)**	53 (17)	100 (14)	65 (11)	92 (12)	75 (3)	14 (1)

The percentage of isolates within each genus exhibiting a given phenotype is shown with the number of isolates tested in parentheses. Little or no adhesion (0/+), medium to high production (++/+++).

**Figure 3:**
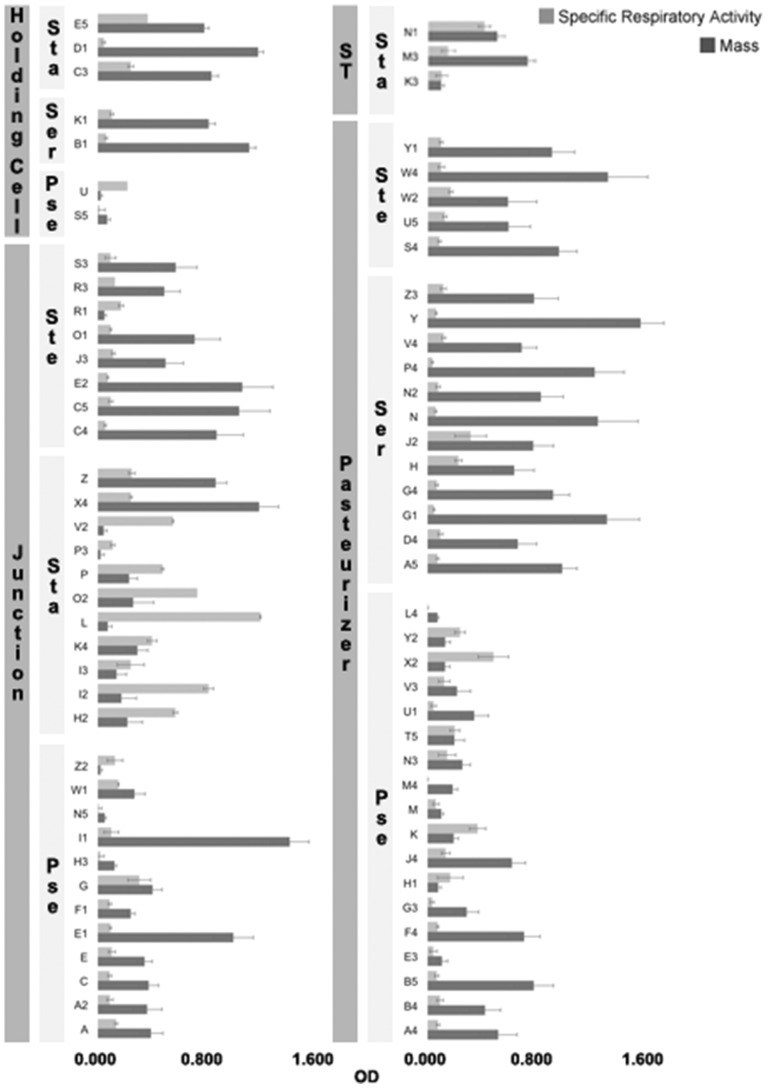
Characterization of the isolates biofilm. Biofilm formation capacity (in TSB) and composition in terms of total mass (Mass) and cells per mass (Specific Respiratory Activity, SRA). Isolates (2 digit labels) sorted by sampling site and genus (ST  =  Storage Tank). Ste - *Stenotrophomonas* spp.; Sta - *Staphylococcus* spp.; Ser - *Serratia* spp.; Pse - *Pseudomonas* spp. Values correspond to the optical density (OD measured (mass - OD at 570 nm; SRA - 570 nm/490 nm).

Each genus had one or a few isolates more capable of forming biofilms with higher mass (Fig. 3). Analyzing each sampled location individually, we observed that in the pasteurizer *Serratia* and *Pseudomonas* spp were the predominant species. We also observed that *Stenotrophomonas* spp. were less abundant, albeit stronger biofilm formers. These should also be the species responsible for the establishment of biofilms in the junction. Nevertheless, an additional but smaller mass contribution should also come from *Pseudomonas* and *Staphylococcus* spp. - only a few of these isolates showed abnormal (high) capacity to produce matrix, in comparison to their relatives (Fig. 3). *Staphylococcus* spp. isolates obtained from the holding cell were extremely active, as indicated by the high specific respiratory activity (SRA) value, translated in a higher ratio of cells to mass. Interestingly, the isolates of the genus isolated from the junction formed biofilms with a lower proportion of metabolically active cells, despite their larger mass. *Pseudomonas* spp. general capacity of forming biofilm was overall much lower than that of *Serratia* spp. isolates, independently of the sampling site. In terms of the cell:mass ratio, *Serratia* spp. seems to dominate (Fig. 3).

## Discussion

Milk processing lines have been previously subjected to studies aiming to characterize their microbial constituents before [18], [19] or after [6], [7], [14], [21] regular disinfection procedures. In this study and contrary to the majority of the previous ones, isolates were identified by 16S *rDNA* sequencing, and not by biochemical [18], [19] and miniaturized kits [25]. In addition to identifying persisting organisms (by culture-based methods), we also characterized the potential for the isolates to adhere and form biofilms, which is directly related with the capacity to persevere in less favourable environments and withstand cleaning treatments. Besides characterizing these isolates as contaminants capable of negatively influencing the intermediate and final products of a processing line while characterizing them, we also assessed their capacity to produce compounds that impair the growth or that kill pathogenic bacteria. Because any processing line offers a wide variety of niches with specific properties where microorganisms can proliferate, we sampled a multitude of loci.

Similarly to previous studies [14], *Pseudomonas* spp. were found to be the most prevalent microorganisms, from those isolated from the milk plant. Species belonging to this genus, particularly *P. fluorescens*, are common inhabitants of milk plants and have also been previously found in this sort of facility after disinfection [14]. These bacteria have been thoroughly described as the major organisms responsible for the degradation of milk properties: protein degradation is revealed by bitterness, and lipases lead to a soapy and rancid flavour [20]. Protease production was wide-spread among different sampling sites with *Pseudomonas* spp. being the most abundant protease producers. Others have reported similar findings, with *P. fluorescens* being the most abundant species of *Pseudomonas,* with 51% of the isolates [19]. Lipase production, under the test conditions, was only observed by one *Serratia* isolate from the pasteurizer and two *Staphylococcus* isolates from the junction.

*Serratia, Stenotrophomas* and *Staphylococcus* spp. were also present, but in lower levels. It has been observed that pathogenic bacteria can also inhabit milk processing plants [20]. For the isolates here obtained, the proxy used for assessment of their pathogenicity was lecithinase secretion capacity. These enzymes have been described as directly related to the pathogenicity of bacteria, since they are involved in the destruction of animal tissue [26]–[28]. Maybe not surprisingly, the genus *Pseudomonas* comprised considerable number of potentially pathogenic bacteria [26], [28]. The absence of *S.* *aureus* (but presence of *S. sciuri*) among the isolates could very well be considered an indication that there were no cases of mastitis around the time of milk collection, or that the sanitation procedure had efficiently removed this species.

As far as we could assess, this was the first study detecting *Serratia* spp. in a milk processing plant, though *Serratia* spp. have been previously described in a vegetable processing plant [10], [29]. Almost half (43%) of the *Serratia* isolates in our study secreted bacteriostatic and/or compounds against *E. coli* and/or *S. aureus*.

Other species sampled from the several devices within the milk processing line were of low prevalence and mostly likely contaminants of animal or feed origin: *Raoultella ornithinolytica, Ochrobactrum grignonense, Rhodococcus erythropolis, Brevibacterium antarcticum, Alcaligenes faecalis* and *Achromobacter xylosoxidans*. It was also observed that the colonization of the different devices was carried out by different organisms, leading to a diverse distribution pattern. The lowest number of colonizers (4) was obtained from the storage tank, which is maintained at low temperature (4°C).

One of the aspects that we felt was lacking in many of the previous studies of this sort was the assessment of the isolates' capacity to form biofilms. The capacity of the isolates to colonize surfaces and establish biofilms is directly related with the prevalence of microorganism post-sanitization [7]. These structures can allow not only for the persistence of contaminants within a location, but can also stand as contamination origin for other locations, by sloughing. This aspect is then of crucial importance for the determination of the Critical Control Points of contamination [13], as part of any HACCP system [6]. In this context, biofilm formed within milk processing line devices can be not only a reservoir of spoilage and pathogenic bacteria, but also a source of downstream biological contamination, contributing to the erosion of devices and products, with consequent economical or health [4] impact. Previous studies have independently reported on the presence of biofilms in areas of difficult reach by traditional disinfection procedures, such as gaskets, crevices and dead ends [1], [2], [30].

The first step of biofilm formation is bacterial adhesion to surfaces. Though most of the isolates were able to adhere to surfaces, their capacity to establish 3-dimensional structures varied with location and genus. *Serratia* spp. stood out as yielding high adhesion capacity and being good biofilm formers, with abundant matrix. Nevertheless, it should be noted that these assays were performed with single species. Biofilms occur naturally as complex communities [1], [2], [30], but lacking understanding of how the sampled communities are organized, single-species growth was characterized. For simplicity of the analysis, it was assumed that species would exhibit the same behaviour pattern in mono and multiculture. Also and for standardization of the results, polyethylene (microtiter plates) was used as the bacterial attachment surface for biofilm formation, despite the bias - if any - this might introduce [31]. The higher capacity of *Stenotrophomonas* from the pasteurizer to form biofilms, in comparison with the other isolates obtained from that same location, could be interpreted as these isolates being responsible for keeping the community together. *Pseudomonas* spp biofilms tended to have a smaller ratio mass:cells and occurred together with species presenting the opposite pattern. It is possible that they would live in a symbiotic relationship, where *Pseudomonas* spp would share the essential siderophores it releases in high quantity, while receiving structural help and physical protection from *Stenotrophomonas* spp. that generally presented the opposite phenotype. It has been previously established that siderophores are a good that can be used as currency between producers and non-producers, as iron is one of the most growth-limiting elements in nature [14]. Unable to secrete their own siderophores, some species use their neighbours - kin or not - to obtain the scarce ferrous iron, required for growth. Siderophores have also been reported to correlate with virulence, in some species [24], [32].

An alternative explanation for the persistence of bacteria on surfaces post-cleansing could be their intrinsic capacity to withstand cleaning and/or disinfection, through acquired resistance [14]. The capacity of bacteria to form biofilms also contributes to the persistence of bacteria, as the secreted polymeric matrix provides them with protection from the surrounding environment, thus from cleansing agents. Even though biofilm removal can eliminate this problem, the difficulty lies in the capacity to effectively and completely remove the biofilm [33]–[36]. The constant abundance of oxygen and nutrients can very well allow for bacteria only mildly affected by the cleaning procedure to fully recover and re-colonize the processing line.

We were interested in looking at these "microbial reservoirs" within food processing plants not only as contributors to the spoilage of the product, but also as source of downstream contamination with potentially hazardous consequences. A variety of microorganisms is indeed capable of resisting sanitation procedures in food preparation facilities, such as the milk processing plant described in this study. These organisms, found within surface-associated biofilms, represent a source of contamination and degradation of the final product. Nevertheless, some have also been found to be unexplored sources of compounds with antimicrobial activity against pathogenic bacteria (*P. aeruginosa* ATCC 10145 and *E. coli* CECT 434, *S. epidermidis* CECT 231, *L. monocytogenes* foodborne isolate and *S. aureus* CECT 976. Thus, with this screening and characterization we intend to encourage scientists to look at contaminants under a different light, as a source of potentially new molecules, of industrial and/or pharmaceutical interest.

## Materials and Methods

### Isolation Procedure

Upon visits to a local milk industry facility (Experimental Station of Paços de Ferreira, Portugal) four sites were aseptically sampled using sterile swabs. For the previous three years, the sampling sites had been subjected to a daily cleaning cycle of diluted nitric acid at 65–75°C, for 30 min, and a concentrated solution of sodium hydroxide, followed by sterile water rinsing. The locations sampled after the regular sanitation procedure were (Fig. 1):

- A storage tank - a removable unit used for transporting milk from the producer to the plant at a stable temperature of 4°C;
- A junction between a storage tank and a transfer pump - for transfer of incoming from the storage tank to the processing line;
- An entry point of an holding cell - precedes the pasteurizer and heats the milk up to a temperature of 63°C;
- The inner side of a plate pasteurizer - this plate pasteurizer had not been opened for an in-depth cleaning/scrubbing in the previous three years.

These sites were chosen seeking a wider diversity of contaminating microorganisms, withstanding different growth conditions.

### Identification of Bacteria and Culture Conditions

The isolation of microrganisms from swabs was performed by successive streaking on Tryptic Soy Agar (TSA) and Skim Milk Agar (SMA). A representative subset of colonies from each plate were picked and streaked onto new plates until complete isolation was achieved. After isolation, the strains were submitted for DNA isolation and 16S *rDNA* sequencing (DNAVision S.A., Belgium). Briefly, for each isolate DNA was purified and the 16S *rDNA* region amplified using specific primers. This resulted in sequences averaging 1.600 base pairs in length, that were then sequenced with 16S *rDNA* primers. Identification of isolates was done by performing homology searches with Basic Local Alignment Search Tool (BLAST) [37] of the 16S *rDNA* genes sequences obtained as previously described. Species names were assigned whenever the degree of homology was higher than 98%.

### Phenotypic Characterization

To evaluate the production of proteases, lipases and lecithinases, the isolates were inoculated in appropriately supplemented media as previously described [19]. Briefly, protease production was assessed by streaking bacteria on 10% SMA and recording the ability to form a degradation. Lipase production was detected by assessing halo formation when strains were grown on Tributyrin Agar: 2.5 g/L meat peptone (Merck - VWR, Portugal), 3.0 g/L yeast extract (Merck - VWR, Portugal), 2.5 g/L Peptone from casein pancreatically digested (Merck), 1% (w/w) tributyrin (Merck - VWR, Portugal) and 12.0 g/L agar powder (Merck), according to the manufacturer. In order to assess the production of lecithinases, 10% egg yolk (Fluka - Sigma, Portugal) was added to Plate Count Agar (Merck - VWR, Portugal) and halo size was recorded [19]. For all three assays, plates were incubated at 25°C for 24–48 hours. Following growth, the digestion degree was subdivided into 4 categories: 0 for digestion absence; + for minimal digestion; ++ for medium and +++ for maximum level of digestion. The screening for siderophore production was performed as previously described [38], using Chrome-Azurol S Agar plates.

### Antimicrobial Activity of Cell-free Spent Media

Each isolate was inoculated in 200 mL of SMB (Skim Milk Broth) and TSB (Tryptic Soy Broth), and allowed to grow for 8 d at 25°C and 140 rpm. Following the incubation period, the cultures were submitted to a 20 min, 15,000 rpm centrifugation at 4°C. The supernatant was filter-sterilized (0.2 µm gyrodiscs, Orange Scientific, USA), divided in aliquots and subsequently kept at −20°C.

Antimicrobial activity of cell-free spent media was assessed on lawns of pathogenic and non-pathogenic bacteria. Gram negative (*P. aeruginosa* ATCC 10145 and *Escherichia coli* CECT 434) and Gram-positive (*S. epidermidis* CECT 231, *L. monocytogenes* foodborne isolate and *S. aureus* CECT 976) bacteria were grown overnight in TSB, at 37°C under shaking conditions (120 rpm). Following the National Committee for Clinical Laboratory Standards [39], lawns of these cultures were laid onto TSA, using cotton swabs, and allowed to air-dry. Ten µL droplets of each culture filtrate were directly applied onto the lawn, and left to dry. The plates were then incubated 24 h at 37°C, and analyzed for halos.

### Biofilm Formation and Analysis

Each isolate was grown in an overnight culture and the optical density (OD_640 nm_) was adjusted to a value of (OD_640 nm_) of 0.4 or about 1.8×10^8^ CFU/mL in TSB, after a washing step with phosphate-buffered saline (5,000 rpm, 4°C, 5 min). 96 well microtiter plates (flat bottom, polystyrene, tissue culture treated, Orange Scientific®) were inoculated with 200 µL of each suspension, and incubated for 24 h, at 25°C (isolates) or 37°C (pathogens) and 140 rpm. Wells containing solely sterile medium were used as control.

The biofilm mass was determined by the crystal violet method [40], [41]. Briefly, biofilms were washed twice with 250 µL/well of phosphate buffer, and left to air-dry, and the remaining biofilm was fixed with an equal amount of 98% methanol (Vaz Pereira, Portugal), for 15 min. Methanol was discarded and plates were allowed to dry prior to the addition of crystal violet (CV) (Gram colour-staining set for microscopy, Merck). After 5 min, CV was discarded and the plates were gently rinsed with running tap water. Once dried, the dye was solubilized with 200 µL of 33% (v/v) glacial acetic acid (Merck, Portugal). The OD of the resulting solution was measured at 570 nm, using a microtiter plate reader (BIO-TEK, Model Synergy HT).

Strain adherence was classified as previously described [41], as non-adherent (0), weakly adherent (+), moderately adherent (++) and strongly adherent (+++). This was determined by calculating the cut-off OD (OD_c_) for the microtiter test as three standard deviations above the mean OD of the negative control and comparing it with the OD of the biofilm of each isolate. For non-adherent bacteria: OD ≤ OD_c_; weakly adherent: OD_c_<OD≤2. OD_c_; moderately adherent: 2.OD_c_<OD≤4. OD_c_ and for strongly adherent 4. OD_c_<OD.

Following Stevens and Olsen [42], the respiratory activity of bacteria within biofilms was indirectly measured using 250 µL/well of a 50 µg/mL sodium 3,3'-[1[(phenylamino)carbonyl]-3,4-tetrazolium]-bis (4-methoxy-6-nitro) benzene sulfonic acid hydrate (XTT) and 10 µg/mL phenazine methosulfate (PMS) solution reaction. Bacteria, in the presence of an electron carrier (PMS), are able to reduce XTT to water-soluble orange formazan. After a three-hour incubation in the dark, the OD of the solution was measured at 490 nm (BIO-TEK, Model Synergy HT). The results from the respiratory activity were expressed per mass: specific respiratory activity (SRA). These were calculated by dividing the OD value of XTT-PMS by the mean OD of the mass, for an indication of the proportion of metabolically active cells within the biofilm. The XTT (Sigma) and PMS (Sigma) solution was freshly prepared when needed, and kept protected from the light at 4°C.

Both the crystal violet staining and the respiratory activity measurement were performed on the controls, for correction of the readings for blank wells.

## Supporting Information

Table S1 **Characterization of microbial isolates from a milk processing line.** Sampling sites: HC - holding cell; JUN - storage tank - transfer pump junction; PAST - pasteurizer; ST - storage tank; Screenings: AM - antimicrobial secretion; CAS - siderophore secretion; ND No growth on the medium; Activity observed: 0 - no activity; 1 - reduced activity; 2 - medium activity; 3 - high activity P - positive; N – negative Bacterial adherence: NA - Non-adherent; + - Weakly adherent; ++ - Moderately adherent; +++ - Strongly adherent
(DOCX)
