# Supplementary material for: Characterization of Contaminants from a Sanitized Milk Processing Plant
Source: PLoS One. 2012 Jun 28;7(6):e40189. doi: 10.1371/journal.pone.0040189 (PMC3386184; doi:10.1371/journal.pone.0040189)
Supplement: Table S1 — Characterization of microbial isolates from a milk processing line. Sampling sites: HC - holding cell; JUN - storage tank - transfer pump junction; PAST - pasteurizer; ST - storage tank; Screenings: AM - antimicrobial secretion; CAS - siderophore secretion; ND No growth on the medium; Activity observed: 0 - no activity; 1 - reduced activity; 2 - medium activity; 3 - high activity P - positive; N – negative Bacterial adherence: NA - Non-adherent; + - Weakly adherent; ++ - Moderately adherent; +++ - Strongly adherent (DOCX) [file pone.0040189.s001.docx]

Supplemental table 1. Characterization of microbial isolates from a dairy processing line.

| **Sampling site** | **Genus/Species** | **Strain** | **AM** | **CAS** | **Enzyme activity** | | **Adherence** |
| --- | --- | --- | --- | --- | --- | --- | --- |
|  |  |  |  |  | **Protease** | **Lecithinase** |  |
| HC | *Alcaligenes faecalis* | F5 | N | 2 | 2 | 0 | ++ |
| HC | *Brevibacterium antarcticum* | S2 | N | 0 | ND | 0 | NA |
| HC | *Pseudomonas mosselii* | S5 | N | 0 | 3 | ND | NA |
| HC | *Pseudomonas mosselii* | U | N | 2 | 3 | ND | NA |
| HC | *Serratia plymuthica* | B1 | P | 1 | 3 | 0 | +++ |
| HC | *Staphylococcus sciuri* | C3 | N | 0 | ND | 0 | +++ |
| HC | *Staphylococcus sciuri* | D1 | N | 0 | ND | 2 | +++ |
| HC | *Staphylococcus sciuri* | E5 | N | 2 | ND | 0 | +++ |
| JUN | *Alcaligenes faecalis* | G5 | N | 3 | 2 | ND | ++ |
| JUN | *Alcaligenes faecalis* | H5 | N | 3 | 1 | ND | ++ |
| JUN | *Brevibacterium antarcticum* | N4 | N | 0 | ND | 0 | +++ |
| JUN | *Ochrobactrum grignonense* | M1 | N | 1 | ND | 0 | +++ |
| JUN | *Pseudomonas fluorescens* | A | N | 2 | ND | ND | ++ |
| JUN | *Pseudomonas fluorescens* | A2 | N | 2 | ND | ND | ++ |
| JUN | *Pseudomonas fluorescens* | C | N | 3 | ND | 2 | ++ |
| JUN | *Pseudomonas fluorescens* | E | N | 2 | ND | ND | ++ |
| JUN | *Pseudomonas fluorescens* | E1 | N | 1 | 3 | ND | +++ |
| JUN | *Pseudomonas fluorescens* | F1 | N | 1 | ND | ND | ++ |
| JUN | *Pseudomonas fluorescens* | G | P | 2 | ND | 3 | ++ |
| JUN | *Pseudomonas fluorescens* | H3 | N | 3 | ND | 3 | + |
| JUN | *Pseudomonas fluorescens* | W1 | N | ND | ND | 3 | ++ |
| JUN | *Pseudomonas fluorescens* | Z2 | N | 2 | ND | 3 | NA |
| JUN | *Pseudomonas psychrophila* | I1 | P | 2 | ND | 2 | +++ |
| JUN | *Pseudomonas putida* | N5 | N | 3 | 0 | ND | NA |
| JUN | *Raoultella ornithinolytica* | W | N | 2 | 1 | ND | +++ |
| JUN | *Rhodococcus erythropolis* | Q4 | N | 1 | 1 | 0 | NA |
| JUN | *Staphylococcus sciuri* | H2 | N | 3 | ND | 0 | + |
| JUN | *Staphylococcus sciuri* | I2 | N | 0 | ND | 0 | + |
| JUN | *Staphylococcus sciuri* | I3 | N | 1 | ND | 0 | + |
| JUN | *Staphylococcus sciuri* | K4 | N | 2 | ND | 2 | ++ |
| JUN | *Staphylococcus sciuri* | L | N | 1 | 3 | 0 | NA |
| JUN | *Staphylococcus sciuri* | O2 | N | 0 | ND | 1 | ++ |
| JUN | *Staphylococcus sciuri* | P | N | 1 | ND | 3 | ++ |
| JUN | *Staphylococcus sciuri* | P3 | N | 1 | 3 | 2 | NA |
| JUN | *Staphylococcus sciuri* | V2 | N | 1 | 3 | 1 | +++ |
| JUN | *Staphylococcus sciuri* | X4 | N | ND | 3 | 0 | +++ |
| JUN | *Staphylococcus sciuri* | Z | N | ND | 3 | 0 | +++ |
| JUN | *Stenotrophomonas maltophilia* | C4 | N | 0 | ND | 0 | +++ |
| JUN | *Stenotrophomonas maltophilia* | C5 | N | ND | 3 | ND | +++ |
| JUN | *Stenotrophomonas maltophilia* | O1 | N | 1 | 3 | ND | +++ |
| JUN | *Stenotrophomonas maltophilia* | R1 | N | 0 | 3 | ND | NA |
| JUN | *Stenotrophomonas maltophilia* | R3 | N | 1 | ND | ND | +++ |
| JUN | *Stenotrophomonas maltophilia* | S3 | N | 1 | 3 | ND | +++ |
| JUN | *Stenotrophomonas maltophilia* | W2 | N | 0 | 3 | ND | +++ |
| JUN | *Stenotrophomonas maltophilia* | Y1 | N | 1 | 3 | 0 | +++ |
| PAST | *Achromobacter xylosoxidans* | L5 | N | 0 | 1 | 0 | ++ |
| PAST | *Ochrobactrum grignonense* | J5 | N | 1 | 3 | 2 | ++ |
| PAST | *Pseudomonas fluorescens* | A4 | P | 2 | ND | ND | +++ |
| PAST | *Pseudomonas fluorescens* | B4 | N | ND | 3 | ND | ++ |
| PAST | *Pseudomonas fluorescens* | E3 | N | 2 | ND | 3 | NA |
| PAST | *Pseudomonas fluorescens* | F4 | N | 1 | 3 | ND | +++ |
| PAST | *Pseudomonas fluorescens* | G3 | P | 2 | ND | ND | ++ |
| PAST | *Pseudomonas fluorescens* | H1 | P | 2 | ND | 3 | NA |
| PAST | *Pseudomonas fluorescens* | J4 | N | 1 | ND | ND | +++ |
| PAST | *Pseudomonas fluorescens* | K | P | 2 | 3 | ND | + |
| PAST | *Pseudomonas fluorescens* | L4 | N | 1 | 3 | 0 | NA |
| PAST | *Pseudomonas fluorescens* | M4 | N | 2 | ND | 3 | + |
| PAST | *Pseudomonas fluorescens* | T5 | P | 3 | 3 | ND | + |
| PAST | *Pseudomonas fluorescens* | V3 | N | 2 | 2 | 2 | + |
| PAST | *Pseudomonas fluorescens* | X2 | N | 2 | ND | 2 | + |
| PAST | *Pseudomonas fluorescens* | Y2 | P | 2 | 3 | ND | + |
| PAST | *Pseudomonas sp.* | B5 | N | 0 | 3 | ND | +++ |
| PAST | *Pseudomonas sp.* | M | N | 1 | 3 | ND | NA |
| PAST | *Pseudomonas sp.* | N3 | P | 1 | 3 | ND | ++ |
| PAST | *Pseudomonas sp.* | U1 | P | 0 | ND | ND | ++ |
| PAST | *Serratia plymuthica* | A5 | P | 2 | 2 | 0 | +++ |
| PAST | *Serratia plymuthica* | D4 | N | 3 | 3 | ND | +++ |
| PAST | *Serratia plymuthica* | J2 | N | 3 | 3 | ND | +++ |
| PAST | *Serratia plymuthica* | N | N | 3 | ND | ND | +++ |
| PAST | *Serratia plymuthica* | N2 | P | 1 | ND | 3 | +++ |
| PAST | *Serratia plymuthica* | P4 | N | 1 | ND | ND | +++ |
| PAST | *Serratia plymuthica* | Y | P | 3 | 1 | 0 | +++ |
| PAST | *Serratia plymuthica* | Z3 | N | ND | 3 | ND | +++ |
| PAST | *Serratia sp.* | V4 | P | 3 | 3 | 0 | +++ |
| PAST | *Stenotrophomonas maltophilia* | E2 | N | ND | 3 | ND | +++ |
| PAST | *Stenotrophomonas maltophilia* | J3 | N | 1 | ND | ND | +++ |
| PAST | *Stenotrophomonas maltophilia* | S4 | N | 1 | ND | ND | +++ |
| PAST | *Stenotrophomonas maltophilia* | U5 | N | 1 | 3 | ND | +++ |
| PAST | *Stenotrophomonas maltophilia* | W4 | N | 1 | 3 | ND | +++ |
| ST | *Alcaligenes faecalis* | T3 | N | 1 | 0 | 3 | + |
| ST | *Staphylococcus sciuri* | K3 | N | 0 | ND | 0 | NA |
| ST | *Staphylococcus sciuri* | M3 | N | 2 | 3 | 0 | +++ |
| ST | *Staphylococcus sciuri* | N1 | N | ND | ND | 0 | +++ |

Sampling sites: HC - holding cell; JUN - storage tank - transfer pump junction; PAST - pasteurizer; ST - storage tank;

Screenings: AM - antimicrobial secretion; CAS - siderophore secretion; ND No growth on the medium;

Activity observed: 0 - no activity; 1 - reduced activity; 2 - medium activity; 3 - high activity

P - positive; N - negative

Bacterial adherence: NA - Non-adherent; + - Weakly adherent; ++ - Moderately adherent; +++ - Strongly adherent
